# Unraveling the morphological complexity of two-dimensional macromolecules

**DOI:** 10.1016/j.patter.2022.100497

**Published:** 2022-04-22

**Authors:** Yingjie Zhao, Jianshu Qin, Shijun Wang, Zhiping Xu

**Affiliations:** 1 Applied Mechanics Laboratory, Department of Engineering Mechanics and Center for Nano and Micro Mechanics, Tsinghua University, Beijing 100084, China

**Keywords:** morphological complexity, two-dimensional macromolecules, molecular simulations, statistical learning, geometrical deformation, topology of contact, lattice distortion, surface interaction

## Abstract

2D macromolecules, such as graphene and graphene oxide, possess a rich spectrum of conformational phases. However, their morphological classification has only been discussed by visual inspection, where the physics of deformation and surface contact cannot be resolved. We employ machine learning methods to address this problem by exploring samples generated by molecular simulations. Features such as metric changes, curvature, conformational anisotropy and surface contact are extracted. Unsupervised learning classifies the morphologies into the quasi-flat, folded, crumpled phases and interphases using geometrical and topological labels or the principal features of the 2D energy map. The results are fed into subsequent supervised learning for phase characterization. The performance of data-driven models is improved notably by integrating the physics of geometrical deformation and topological contact. The classification and feature extraction characterize the microstructures of their condensed phases and the molecular processes of adsorption and transport, comprehending the processing-microstructures-performance relation in applications.

## Highlights

• Morphology of 2D macromolecules are classified into four phases
• Data-driven models capture physics and topology beyond the geometry
• Condensed-phase properties are understood by the features extracted

## The bigger picture

Resolving morphological complexity of macromolecules is the stepping stone to the design and fabrication of high-performance, multi-functional materials and to understanding the soft matter behaviors in biology and engineering. To extract the physics of lattice distortion and surface contact beyond the conformation is critical, yet challenging. Here, we show that, by labeling the simulation data using the 2D map of potential energies, the 3D geometry, and the topology of contact, morphological classification can be achieved with high accuracy. The well-trained model can be used to decipher the microstructural complexity using simulation or experimental data, which may include the geometrical representation only. This data-driven approach extracts the key geometrical and topological features of 2D macromolecules that are directly responsible for the material performance in relevant applications and can be extended to study other complex surfaces such as red blood cells and the brain.

## Graphical abstract

**Figure:**
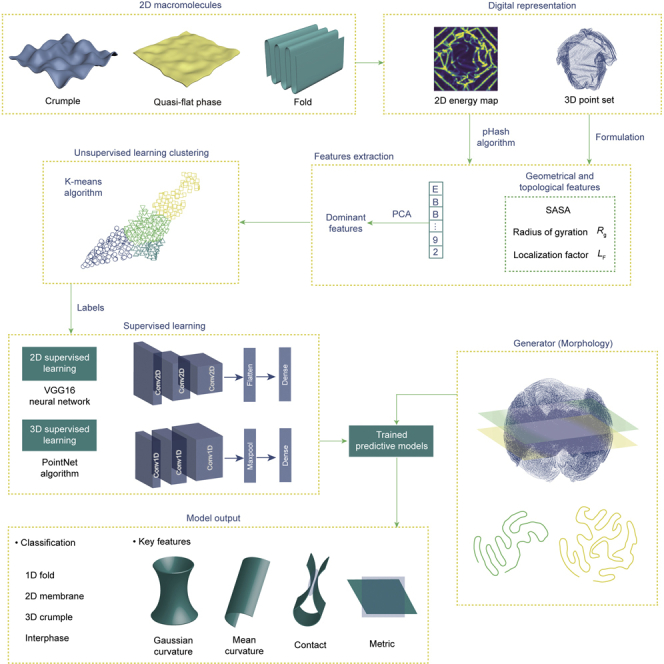

## Teaser

Morphology of 2D macromolecules defines the microstructures of their condensed phases and the processing-microstructures-properties relationship of their macroscopic assemblies. Zhao et al. integrate molecular simulations and statistical-learning methods to classify the 2D macromolecules into four morphological phases. Beyond the conventional approach using 3D geometry, the physics of lattice distortion and the topology of surface contact are included in the models. The key features correlated with the morphological complexity and material properties of 2D macromolecules are revealed.

## Introduction

**Figure 1:**
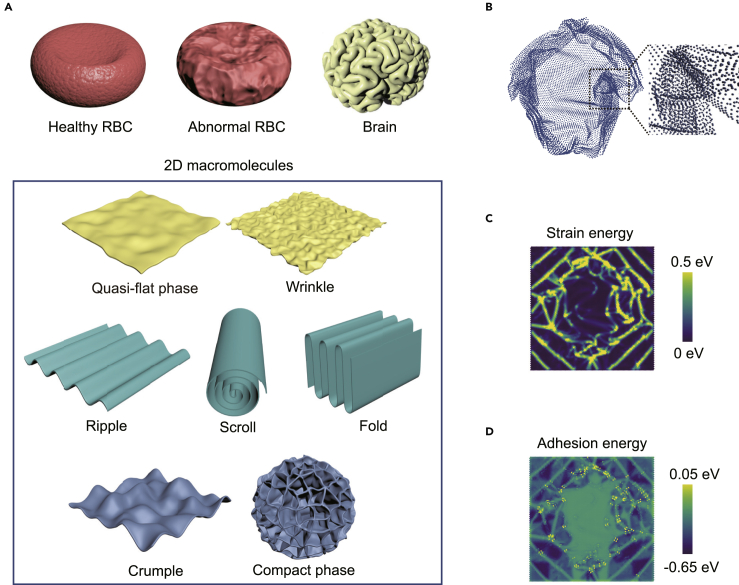
Morphological phases of 2D macromolecules (A) Phases identified from theoretical and experimental studies, which include the flat,^6^^,^^13^^,^^14^ quasi-flat,^9^^,^^18^ rippled,^19^ wrinkled,^20^ folded,^13^^,^^15^^,^^16^^,^^23^ scroll,^21^^,^^22^ crumpled,^10^^,^^12^^,^^14^^,^^17^^,^^18^ and compact^13^^,^^14^ phases. Similar morphological complexity can be found in the red blood cells (RBCs)^24^ and the brain.^25^ These phases are classified into the quasi-flat, folded, and crumpled phases in this work, as indicated by the colors. (B–D) Digital representations through the 3D map of conformation (B), the 2D map of strain energy (C), and adhesion energy (D). The 2D maps of strain and surface adhesion are strongly correlated due to the physics of mechanical equilibrium (Equation 1).

Polymerized linear and nonlinear molecules with repeating one-dimensional (1D) subunits feature a variety of geometrical forms such as 1D chains, 2D membranes, and 3D dendrimers, of which the morphology is a key determinant of the material properties.^1^ 2D macromolecules possess an even richer spectrum of conformational complexity. The competition between the entropy gain caused by thermal corrugation and the enthalpic penalty attributed to the shear and bending resistance governs their conformational evolution.^2^ The relation between their morphological behaviors and size, bending stiffness, and surface interaction were discussed through the conformational scaling laws.^3^, ^4^, ^5^, ^6^, ^7^, ^8^ Numerical simulations using the self-penetrable phantom model of tethered membranes confirm the stability of quasi-flat conformation and revealed the crumpling transition at high temperature.^9^, ^10^, ^11^ Considering the effects of self-avoiding, bending resistance, and surface interaction, simulations of more realistic models predict flat, rippled, wrinkled, crumpled, folded, scrolled, and compact phases,^6^^,^^12^, ^13^, ^14^, ^15^, ^16^, ^17^, ^18^ which are validated by the experimental studies^19^, ^20^, ^21^, ^22^, ^23^ (Figure 1A).

Morphology of 2D macromolecules plays an important role in defining the microstructures of their macroscopic assemblies in forms of fibers and films, as well as their mechanical, transport, and functional properties.^26^, ^27^, ^28^ For example, plasticization spinning strategy converts the wrinkled conformation of graphene to the flat one and enlarges the crystallite domains, thus enhancing the mechanical properties of graphene fibers.^29^ Microfluidic design regulates the alignment and orientation of graphene sheets and improves the transport properties of fibers.^30^ Graphene films assembled from the flat, folded, and crumpled phases exhibit an improved hydrophobicity in sequence.^23^ Solvent-mediated topography regulation triggers reversible fusion-fission transitions of graphene fibers, where the solvent modulates morphologies of the shell and interface in the fibers, switching between the wrinkled and spread phases.^31^

Folded or crumpled phases of 2D macromolecules also define the adsorption and transport processes of ions and molecules in these condensed phases, which are the key to develop relevant energy and environment applications. For example, electrodes of folded graphene provide continuous transport pathways with high electron or ion mobility,^32^ where the crumpled phases demonstrate high specific surface area pore volume and excellent capacitance.^33^ Crumpled graphene balls possess meso- and micro-pores as well as stacking-resistant structures, exhibiting efficient micro-pollutant absorption from water.^34^ Rationalizing the conformational map is thus of critical importance to understand and control the microstructures and functions of 2D macromolecules and their assemblies.^28^^,^^35^

Although ample morphological phases of 2D macromolecules were reported in previous studies, their classification has been limited by visual inspection of the geometry. In this work, we use machine learning techniques to discriminate the morphological phases of 2D macromolecules. Data-driven statistical-learning techniques developed for pattern recognition and prediction^36^, ^37^, ^38^ have been applied in the materials sciences.^39^^,^^40^ Unsupervised and supervised learning studies identify the distinct polymer states.^41^, ^42^, ^43^ For 2D macromolecules, unsupervised learning was used to classify graphene oxide (GO) according to the chemistry (the C/O ratio) and morphology (the mean size of flakes), which were determined by X-ray photoelectron spectroscopy and scanning electron microscopy analysis, respectively.^44^ Supervised learning recognizes nanobubbles in graphene from the electronic density of states spectra, and predicts the height and width of nanobubbles.^45^ Statistical-learning methods combining unsupervised and supervised learning have been utilized in the conformational recognition of molecules and polymers, and the determination of phase transition.^42^^,^^46^^,^^47^ Self-supervised learning was used to embed geometrical features into the graph neural network to assist in the molecular conformational identification and property prediction.^46^ The pre-training process utilizes abundant unlabeled samples to learn and import the geometrical features into the neural network, and the fine-tune process uses a handful of labeled samples to perform the recognition and property prediction tasks.^46^ The confusion scheme trains models with data that are deliberately labeled incorrectly, and the phase transition can be determined according to the performance of the models trained with different labels.^47^ Following this approach, the configurations of the polymers were recognized, and the critical energies of phase transition were determined.^42^ We thus integrate unsupervised and supervised techniques to provide a tool that can be used to discriminate the conformational phases of 2D macromolecules and offer insights into the transition between them.

Notably, in addition to the geometry of 2D macromolecules, the lattice distortion and topology of surface contact are also of vital importance to understand the microstructures-performance relationship. However, this physics cannot be extracted from the 3D conformation obtained from, for example, experimental computed tomography. To address this issue, we perform coarse-grained molecular dynamics (CGMD) simulations to generate macromolecular structures of graphene and the physics of molecular interaction. Unsupervised learning is conducted based on the features extracted from the energy of strain and adhesion, or the 3D geometry and topology of the contact. The model trained can be used in supervised learning for morphological recognition and classification using simulation or experimental data. This combined approach allows the physics behind the observable geometrical and topological characteristics to be included in the discussion on the morphological complexity, and the assessment of their significance. The results lay the ground for the understanding of processing-microstructures relationships of 2D macromolecules, and the design principles of macroscopic assemblies with outstanding performance and functions.

## Results

### Digital representation of 2D macromolecules

We generate 2,484 samples of conformation from the CGMD simulations, as well as the potential energy of each atom, bond, dihedrals, and non-bonding interacting pairs (see experimental procedures for details). The initial configuration of graphene is a flat square sheet with lateral size *L* of 100 nm. We use isotropic, spherical and anisotropic, and cylindrical confinement, as well as their linear combination, to trigger conformational changes of the 2D macromolecules.^17^^,^^23^^,^^48^ By further exploring the parameter space spanned by the temperature and the bending stiffness, the simulation results cover a large subspace of the morphological phases ranging from 1D to 3D.

2D macromolecules can be represented directly using the point set from numerical simulations or experimental tomography. This approach captures the full geometrical information including the curvature and a distance map in the 3D Euclidean space. However, although the ridges or vertices can be identified by their geometrical features, the metric changes in the basal plane and out-of-plane bending of 2D macromolecules cannot be represented in the point set without a reference geometry. Moreover, the physics of surface contact cannot be extracted from a point-set representation, where the intramolecular bonding network and surface contact cannot be distinguished (Figure 1B).

The conformation is also analyzed through the 2D map of potential energy to extract key conformational features,^23^ which captures the physics of lattice distortion (metric changes, bending) and surface adhesion implicitly from the energy of the bonded and non-bonding interaction (see experimental procedures). Mapping the atomic positions into the initial planar configuration of 2D macromolecules, the network structure of ridges and vertices can be visualized from the map of strain energy (Figure 1C). Ridges are created by out-of-plane bending, while the vertices accommodate in-plane deformation along with bending. Physical contact forms between regions of the 2D macromolecules through the map of surface adhesion (Figure 1D). However, the topological information is missing in the 2D representation, which could be measured by the distance map in the 2D manifold, which is constructed from the initial configuration. The combination of the 3D point set and the 2D energy map could and should thus be combined to understand the physics behind the geometry and topology of macromolecules.

The conformation of 2D macromolecules is determined by the competition between the resistance to elastic deformation and surface interaction that could be adhesion or steric repulsion. The geometry of the 2D manifold and the topology of contact thus are closely tied to the deformation and surface interaction. Thermal fluctuation also plays a role, especially in the solution environment, in triggering the morphological changes. The potential energy of 2D macromolecules can be modeled through the generalized Helfrich functional^2^

(Equation 1) $$U=\int \text{d}S\left[\frac{1}{2}\kappa {\left(2H-c_{0}\right)}^{2}+\bar{\kappa }K\right]+2\gamma S_{\text{c}}\text{,}$$

where *S* is the surface area. *H* and *K* are the mean and Gaussian curvature, respectively. κ is the bending rigidity, $\bar{\kappa }$ is the Gaussian rigidity, and the extrinsic geometry term $\bar{\kappa }K$ measures the coupling between out-of-plane bending and in-plane deformation. *c*_0_ is the spontaneous curvature induced by topological defects embedded in the 2D macromolecules, which is zero here.^49^ γ is the surface energy density determined by the van der Waals or electrostatic interaction, and *S*_*c*_ is the area of contact. This functional, in combination with the entropy term, defines the geometry and topology of 2D macromolecules in 3D space. The physics behind the conformational phases can thus be extracted from the potential energy of lattice distortion and surface contact, as well as the geometrical and topological measures of the conformation.

### Feature extraction

**Figure 2:**
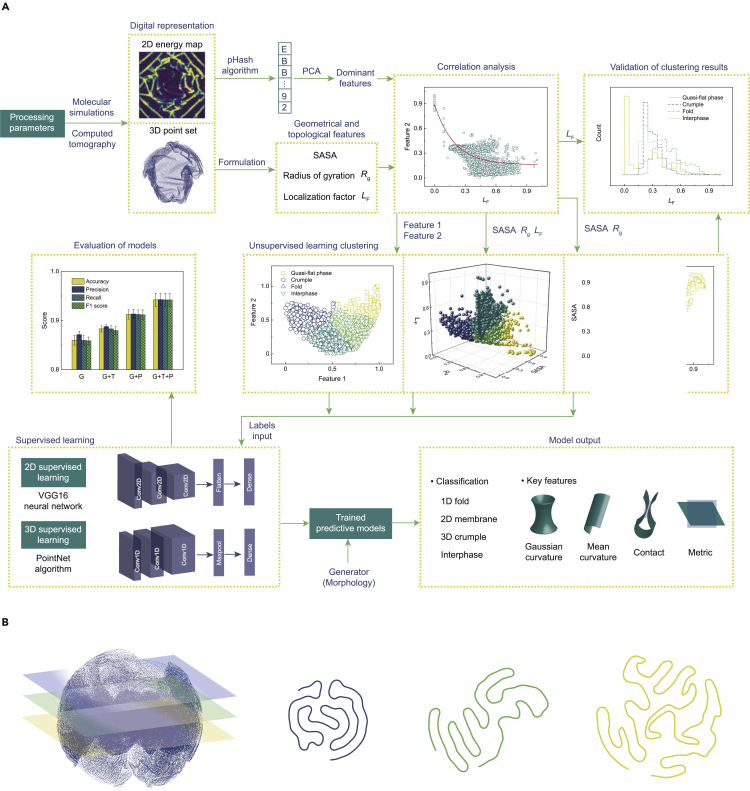
Machine learning procedures (A) Flowchart of conformational recognition and features extraction for 2D macromolecules. (B) Illustration of the computed tomography data of 2D macromolecules.

The flowchart of conformational classification and feature extraction in this work is illustrated in Figure 2. The solvent-accessible surface area (SASA) and radius of gyration (*R*_g_) are the two key geometrical features measuring the surface exposure and the compactness of conformation, respectively (see experimental procedures.^17^^,^^48^ Surface contact is a topological feature that modulates the transport processes through the open spaces embedded in the condensed phases, such as the folds and crumples. A localization factor (*L*_F_) is defined to characterize the degree of localization for surface contact from the 2D distance map, discriminating the local and long-range modes of contact (see experimental procedures). The combination of SASA, *R*_g_, and *L*_F_ provides multi-resolution characterization of the 3D geometry and topology of contact for 2D macromolecules, which are used for labeling in the unsupervised learning.

Features in the 2D energy map of strain and adhesion are extracted by the perceptual Hash (pHash) algorithm^50^ and principal-component analysis (PCA).^51^ The pHash algorithm converts the pixels information of images into a string of fingerprints for comparison. PCA further reduces the dimension of these fingerprints to yield the dominant features, the physics of which can be discussed through comparison with the characteristics evaluated directly from the 3D point set.

**Figure 3:**
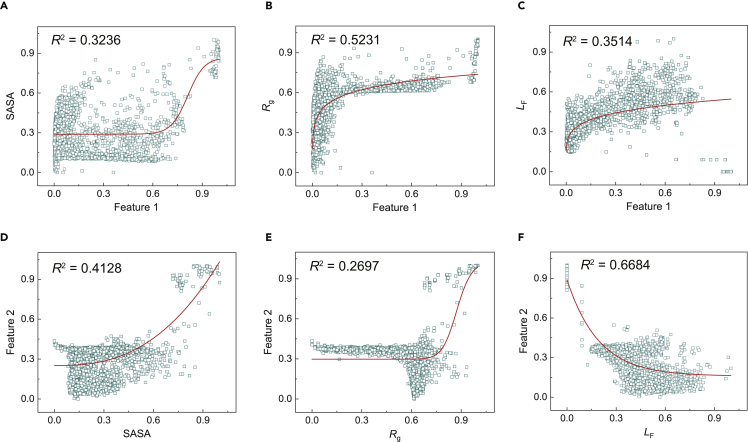
Correlation between features extracted from the 2D map of strain energy and morphological parameters extracted from the 3D point set (A–C) The correlation between the first principal feature (P1) and nondimensionalized values of SASA, *R*_g_, and *L*_F_. (D–F) The correlation between the second principal feature (P2) and nondimensionalized values of SASA, *R*_g_, and *L*_F_.

Correlation between the first and second principal features (P1 and P2, respectively) extracted from the 2D map of strain energy and SASA, *R*_g_, *L*_F_ measured from the 3D conformation are summarized in Figure 3. The results show that P1 has the strongest correlation with *R*_g_ (Figure 3B), and P2 is most relevant to *L*_F_ (Figure 3F), which suggests that P1 captures the global shrinkage of the morphology, while P2 measures the surface contact. SASA is more relevant to P2 than P1 (Figures 3A and 3D). As *L*_F_ has the largest correlation factor with the principal features (Figure 3F), we use SASA and *R*_g_ for unsupervised classification of the conformation, and *L*_F_ for the validation. We also analyze the energy map of surface adhesion, which is highly correlated with the strain energy map (Figure S1). For this reason, our following discussion is limited to the 2D map of strain energy.

### Statistical learning

**Figure 4:**
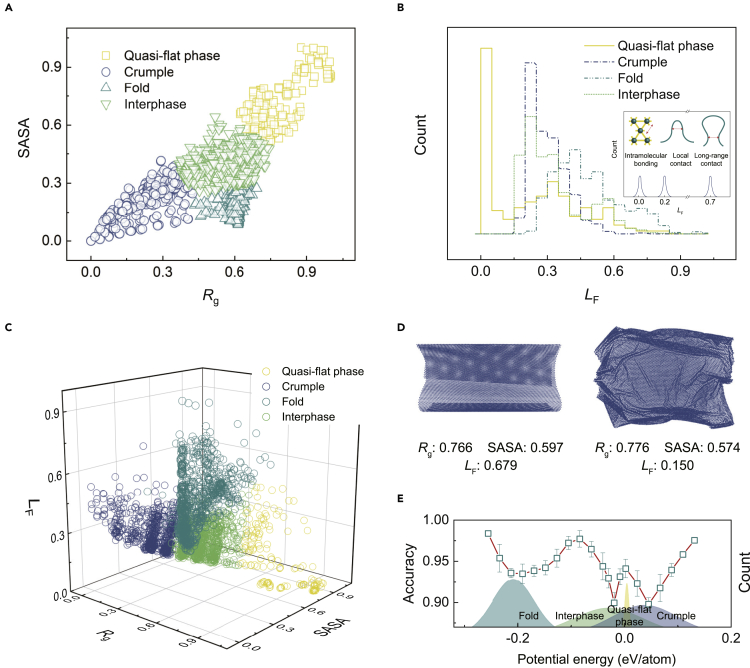
Classification of morphological phases from unsupervised learning (A) Clustering of samples in the space spanned by the normalized values of SASA and *R*_g_. (B) Distribution of normalized *L*_F_, which is defined to capture the bimodal feature of the intramolecular bonding network and (local, long range) surface contact (see the inset for details), respectively. (C) Clustering using SASA, *R*_g_, and *L*_F_ labels. (D) Two samples with similar SASA and *R*_g_ values but different *L*_F_. (E) The distribution of morphological phases in the space of potential energy. The accuracy of prediction in the confusion scheme is used to identify the possible transition states from the peaks, while the valleys are the well-identified phases. The standard deviation is reported in the error bars.

Unsupervised learning based on the extracted features (SASA, $R_{\text{g}}$, $L_{\text{F}}$) are performed to label the data. We first use the K-means algorithm^52^ to classify the conformation in the parameter space of SASA and *R*_g_ only. Four classes are identified as quasi-flat, crumpled, and folded phases, as well as the interphases (Figure 4A). Quasi-flat phases have large SASA and *R*_g_ values for high surface exposure and low shrinkage. Folded phases have a relatively larger value of *R*_g_ than that of the crumples since anisotropic folds only contract in one direction, while isotropic crumples shrink in all directions. The interphases are located between these three well-defined phases. The distribution of *L*_F_ is calculated to validate the clustering-based classification (Figure 4B). In the quasi-flat phases, *L*_F_ shows a peak at the characteristic length scale set by the intramolecular bonding network since no contact is formed. The peak of *L*_F_ of the folded phase has the largest value, indicating that the contact has a long-range nature, while the crumple has a peak at the intermediate distance, and the contact is local. The value of *L*_F_ for the interphases resides between the other three phases, displaying the nature of transition states.

The effect of contact topology is further assessed by including *L*_F_ as one of the labels in addition to SASA and *R*_g_. The results show that the information of topological contact improves the physical significance of clustering, which is of crucial importance for the transport and adsorption processes in the condensed phases or assemblies of 2D macromolecules. For example, we find two samples of the same size with different morphologies but similar SASA and *R*_g_ values (Figure 4D). Their *L*_F_ values, however, show discrepancy. Geometrical clustering classifies these two samples as quasi-flat phase (Figure 4A), but the topological consideration by adding *L*_F_ in the labeling process corrects the prediction by recognizing one of them with the larger value of *L*_F_ to be a fold (Figure 4C).

The energy landscape of morphological phases defines the richness of the morphological phases in thermal equilibrium according to the Boltzmann factor $\text{exp}\left(-k_{\text{B}}T\right)$ (Figures 4E and S2). The crumples and folds own high and low potential energies for their strong lattice distortion and surface adhesion, respectively, and those of the quasi-flat phases or interphases are between them. The path of transition between these phases can be interred from the confusion scheme, where the accuracy of prediction at specific potential energy measures the likelihood of the corresponding morphology as a transitional one (see experimental procedures).^47^ Comparing the accuracy with the distribution of morphological phases over the energy space suggests that the interphases could bridge the crumples and folds, while the quasi-flat phases and some of the interphases show features of transition states (Figure 4E). This result agrees with the experimental finding that direct transition between the crumples and folds is prohibited by the symmetry and should proceed through intermediate phases such as the quasi-flat one.^23^

Supervised learning is carried out using the labels obtained from unsupervised learning to recognize the conformation of 2D macromolecules represented by the 2D map of strain energy or the 3D point set. The labeling using SASA and $R_{\text{g}}$ contains geometrical (G) features only (Figure 4A), and that with $L_{\text{F}}$ includes both geometrical (G) and topological (T) information (Figure 4C). For the representations of macromolecular conformation, the 3D point set comprises the geometrical features, while the 2D energy map includes the physical characteristics of lattice distortion and deformation (P). The combination of labeling and digital representations thus produces four models, which are: geometrical labeling and 3D point-set representation (the G model), geometrical and topological labeling and 3D point-set representation (the G + T model), geometrical labeling and 2D energy-map representation (the G + P model), geometrical and topological labeling and 2D energy-map representation (the G + T + P model). Using these models, we randomly sample the data at a ratio of 0.7:0.15:0.15 for the training, validation, and test sets. Data in the training and validation sets are used for training, and the validation set is also used to adjust the hyperparameters. Samples in the test set are used to evaluate the performance of the well-trained model.

**Figure 5:**
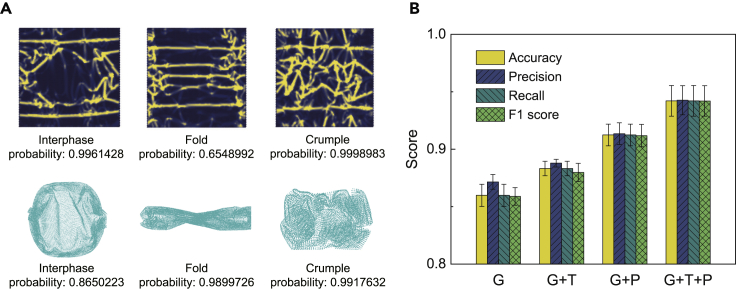
Predictions from statistical learning (A) Phases predicted by the well-trained supervised model using SASA and *R*_g_ as the labels and the corresponding prediction probability. The energy heat images are the maps of strain energy. (B) Evaluation of the statistical-learning models. The standard deviation is reported in the error bars.

The 2D map of strain energy is explored by using the VGG16 neural network.^53^^,^^54^ After training, samples in the test set are used as the input for prediction. The output includes the classified phases and their probabilities (Figure 5A). The confidence of prediction for a specific class is scored by the probability. The 3D morphological map is studied using the PointNet algorithm.^55^ The classification and the corresponding probability of prediction are summarized (Figure 5A). We use the metrics of accuracy, precision, recall, and F1 score to evaluate the models (see experimental procedures). The metric scores of different models are summarized in Table S1. We find that the geometrical features possess a fundamental contribution to all the models. The topological and physical information notably improves the performance of model (Figure 5B). The G + T + P model with the geometrical, topological, and physical characteristics demonstrates the best performance (Figure 5B). We also label the data using the principal features extracted from the 2D energy map (Figures S3A and S3B). The performance of these models is not as competitive as the G + T + P model (Figure S3C). By further considering that extracting SASA, *R*_g_, and *L*_F_ is more convenient than that for the principal features, the usage of direct geometrical and topological labels in unsupervised clustering is preferred in practice.

## Discussion

### 2D versus 3D supervised learning

The 2D map of the potential energy identifies the ridges, vertices, and surface adhesion, but the metric changes and curvature of geometry and the topology of surface contact are only implicitly considered through the energy terms. For the true positives, the higher probability of prediction from 2D supervised learning using the G + P model indicates that physical discrimination through the energy map is more feasible, while geometrical recognition is more convenient in 3D by using the G model (Figure 5A).

Data failing in the prediction from labels in the unsupervised learning is dominated by the interphases with the nature of transition states (Figure S4A). The false negatives of quasi-flat and crumpled phases are less significant in 2D learning, since the lattice distortion can be recognized. The quasi-flat phase has a sparse network of ridges and vertices, while the crumpled phase has a dense one. The false negatives of folds and the interphases are less significant in 3D learning as the geometrical feature is more significant. The fold has a distinct 1D anisotropic feature, while the interphases have mild characteristics in geometry compared with the quasi-flat, folded, and crumpled phases.

### Unsupervised and supervised learning

False predictions may be attributed to poor labeling from unsupervised learning, the results of which are used as the input for supervised learning. For example, there is a sample labeled as the interphases, but predicted as a fold by both 2D (G + P) and 3D (G) supervised learning (Figure S4B). The normalized value of *L*_F_ for this conformation is 0.48108, which aligns with the feature of folds (Figure S4B). The supervised learning thus can outperform unsupervised learning by correcting poor labeling, although unsupervised learning can also be improved by defining more suitable features for extraction (Figure S5). The maximum score achieved by the G + P + T model is 0.9515, which is limited by the physics of labels chosen (SASA, *R*_g_, and *L*_F_). The graph neural networks that implant the topological information into the structure of neural networks may be used for improvement.^56^

### Surface interaction

**Figure 6:**
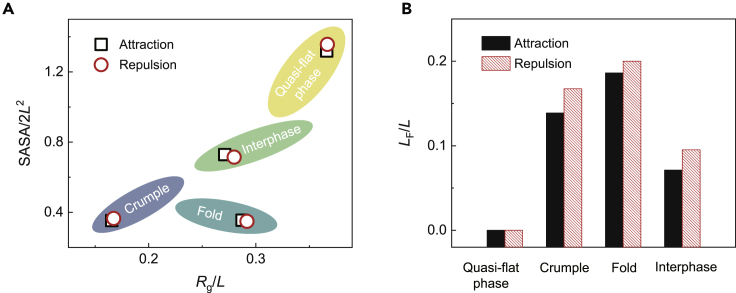
Effects of surface interaction (A) The distribution of samples with attractive and repulsive surface interaction in the parameters space of SASA and *R*_*g*_, which shows minor changes. (B) *L*_F_ calculated for samples with attractive and repulsive surface interaction, which shows significant dependence.

Surface contact in 2D macromolecules can be regulated by solvent or surface modification. For example, flat GO remains stable in the solution with low concentrations of dimethylformamide (DMF), while folded phases are identified in the solution with divalent Ca^2+^ ions due to the short-range attraction between GO, and crumpling occurs in the hydrazine (N_2_H_4_) solution as a result of the long-range attraction after reduction.^23^ Changes in the surface charge density trigger the transition from nanomembranes to nanoscrolls, which reduce the electrostatic potential barrier of nucleation and electrostatic repulsion during the process of scrolling.^22^ The reversibility of phase transitions between the flat phase and folds or crumples depends on the nature of surface interaction. Surface adhesion yields an enthalpic penalty for the process of unfolding or uncrumpling, while repulsion can drive these reverse processes as the boundary constraints are released. We explore the effects of surface interaction by tuning the nature of interaction from being attractive to repulsive in the simulations. The values of SASA, *R*_g_, and *L*_F_ are nondimensionalized by 2*L*^2^ and *L*, which are the surface area and lateral size of the flat square sheet, respectively. The results using the G model show that, SASA and *R*_g_ of 2D macromolecules with repulsive surface interaction are similar as those measured with attraction (Figure 6A). However, the *L*_F_ value increases from attraction to repulsion, indicating the shift from local to long range (Figure 6B).

### The size effects

The data used for morphological classification in this work is limited by the size of simulated models. The size effects can be assessed through a dimensionless measure of *L*/*L*_*p*_, where *L*_*p*_ is the 2D persistence length,$L_{\text{p}}=\xi \text{exp}\left(2\pi \kappa /k_{\text{B}}T\right)$, ξ is the short distance cutoff (the lattice constant), and $k_{\text{B}}$ is the Boltzmann constant.^2^ For $L\;≲\;L_{\text{p}}$, the 2D macromolecules behave as rigid or elastic sheets. Our simulations are focused on models with $L>L_{\text{p}}$ and L≫d, where *d* is the spacing of the surface contact, which is 0.335 nm for graphene and 0.6−0.7 nm for GO. By tuning the values of κ and *T* (see experimental procedures), we modify the value of *L*_*p*_, and generate a rich spectrum of conformational phases (Figure S6A). We find that the values of SASA and *R*_g_ are bounded by the values of the 1D cylindrical, 2D flat, and 3D spherical phases. Moreover, the bounds obtained for samples with *L* = 50, 100, and 500 nm are close (Figure S6B), suggesting that this size effect is not a major issue in the context of discussion here. However, the geometrical features, such as the length of folds and the area of contact, are physically limited by the value of *L* chosen here for the consideration of computational costs.

### The completeness of data

The performance of phase recognition here is limited by the space of conformation generated by our molecular simulations where spatial confinement is enforced to trigger the phase changes. The correlation between the resulted phases and the conditions of confinement shows that crumples and folds are mainly generated by the spherical and cylindrical confinement, respectively, while quasi-flat phases and interphases can be obtained under spherical or cylindrical confinement or their combination (Figure S8A). The effect of temperature in the range of 300−900K is not significant since the kinetic energy is much lower than the energy corrugation during the phase changes (Figure S8B). At high temperature, changes in the covalent bond network, such as the sp^2^-sp^3^ transition, may be activated, which could implant lattice imperfections and crosslinks at the contact. The behaviors of the morphological phases generated by spatial confinement can be considered as enthalpic, while the entropic effects are negligible. In the solvent, however, the entropic contrition to the free energy could be significant, especially for the quasi-flat phases and interphases. These results suggest that our dataset can be expanded, for example, by performing long-time equilibrium simulations, or non-equilibrium ones using the free energy techniques,^57^ which are crucial for accurate identification of the paths and energy barriers of phase transitions, although the procedure could be technically challenging and computationally costly.

### Conclusion

To summarize, we utilize machine learning techniques to classify the morphological phases of 2D macromolecules through the 2D map of lattice distortion, surface adhesion, and 3D conformation. SASA, *R*_g_, and *L*_F_ are defined as the key conformational measure for the surface exposure, compactness, anisotropy, and surface contact, which are compared with the principal features extracted from the 2D map of the potential energy to understand the physics behind the morphological complexity. Unsupervised learning clusters the samples based on their geometrical and topological features, and provides the labels needed in subsequent supervised learning. 2D supervised learning identifies a variety of morphological phases from the potential energy of lattice distortion and surface adhesion. 3D supervised learning completes the discrimination by extracting the geometrical and topological information with a distance map in the 2D manifold supplied.

The well-trained models established with the geometrical, topological, and physical information can be used for recognition and classification of the simulation or experimental data, which may consist of geometrical information only, for practical consideration. The model can be applied to the assemblies of multiple 2D macromolecules, and takes the advantage in the identification of defects, which may create localized lattice distortion and modify the interaction between different regions of the 2D macromolecules, resulting in additional features of geometrical deformation and topological contact (Figure S7). This work thus lays the ground for the understanding of the microstructures and material properties of 2D macromolecules in their condensed phases or macroscopic assemblies, and could be extended to other complex surfaces in, for example, the red blood cells^24^ and the brain.^25^ Our study also suggests that a theoretical description to characterize the morphology of 2D macromolecules should include topological features, such as the local and long-range contacts, in addition to the geometrical representation, which results from the competition between the deformation and surface interaction.

## Experimental procedures

### Resource availability

#### Lead contact

Request for information and resources used in this article should be addressed to Dr. Zhiping Xu (xuzp@tsinghua.edu.cn).

#### Materials availability

There were no physical materials associated with this study.

### Molecular simulations

We use a hexagonal lattice to construct the coarse-grained (CG) models of 2D macromolecules.^58^ The atoms are clustered into beads with equal masses. The bonding interaction between bonded beads is modeled as a linear elastic spring with stiffness $k_{\text{s}}=\left(3^{1/2}/2\right)Yt$, where *Y* is the Young’s modulus and *t* is the thickness. The in-plane elastic energy is thus $U_{\text{s}}=k_{\text{s}}{\left(r-r_{0}\right)}^{2}/2$, where *r* is the bond length with an equilibrium value of *r*_0_. The bending resistance is modeled as a harmonic dihedral with stiffness $k_{\text{b}}=\left(2/\sqrt{3}\right)\kappa$, where κ is the bending stiffness.^59^ The out-of-plane bending energy is $U_{\text{b}}=k_{\text{b}}\left(1+\text{cos}\varphi \right)$, where *φ* is the angle of dihedral with an equilibrium value of $\varphi_{0}=\pi$. The non-bonded interaction between the beads is modeled by the Lennard-Jones 12−6 potential $U_{\text{c}}=4\varepsilon \left[{\left(\sigma /r\right)}^{12}-{\left(\sigma /r\right)}^{6}\right]$ with the 1−2, 1−3, and 1−4 neighbor exclusion enforced, where the parameters σ and ε are fitted through the spacing at the contact and the cohesive energy.^60^ We use the force field parameters developed for GO as the reference, and tune $k_{\text{b}}$ and the cutoff distance *r*_*c*_ in evaluating *U*_*c*_ for the bending stiffness and surface interaction (*r*_*c*_ = 2.5*σ*) from attraction to repulsion ($r_{\text{c}}=2^{\frac{1}{6}}\sigma$), respectively. The parameters of the CG force field used in this study are summarized in Table S2, which can be further modified for generation to other for 2D macromolecules.

A large-scale atomic/molecular massively parallel simulator was used to perform all CGMD simulations.^61^ The initial conformation of 2D macromolecules is flat, and the lateral size *L* is 100 nm if not specified otherwise. Conformational changes are driven by applying spherical, cylindrical, or combined linear elastic constraints. The constraints with a harmonic spring move slowly to interact with the beads representing the 2D macromolecules, triggering the conformational transition. The spring stiffness is set to 20 kcal/mol, and the constant speed of constraints is defined by the compression ratio *R* and duration τ. We generate 2,484 morphological phases by adjusting the temperature in the range of 300−900 K, the bending stiffness between 1 and 60 kcal/mol, and the conditions of constraint (mode, speed, ratio of compression, and duration). The compression ratios of spherical and cylindrical constraints are R=0.4−0.9 and 0.15−0.9, respectively, where the lower values correspond to the compact limits. The duration of compression is set to τ=0.5−2 ns. A Langevin thermostat is used for temperature control and to include the implicit solvent effect. The time step is 1 fs, which assures the stability of the numerical integration.

### SASA, *R*_g_, and *L*_F_

SASA measures the surface area of graphene that is accessible to a solvent, which is calculated using the Shrake-Rupley algorithm,^62^ where a bead of probe with a radius of 2.5 nm is chosen.^63^

The radius of gyration tensor S is defined as

(Equation 2) $$S=\frac{1}{N}\sum_{i=1}^{N}\left[\begin{array}{lll} {\left(x_{i}-x_{\text{c}}\right)}^{2} & \left(x_{i}-x_{\text{c}}\right)\left(y_{i}-y_{\text{c}}\right) & \left(x_{i}-x_{\text{c}}\right)\left(z_{i}-z_{\text{c}}\right)\\ \left(y_{i}-y_{\text{c}}\right)\left(x_{i}-x_{\text{c}}\right) & {\left(y_{i}-y_{\text{c}}\right)}^{2} & \left(y_{i}-y_{\text{c}}\right)\left(z_{i}-z_{\text{c}}\right)\\ \left(z_{i}-z_{\text{c}}\right)\left(x_{i}-x_{\text{c}}\right) & \left(z_{i}-z_{\text{c}}\right)\left(y_{i}-y_{\text{c}}\right) & {\left(z_{i}-z_{\text{c}}\right)}^{2} \end{array}\right]\text{,}$$

where *N* is the number of CG beads, *r*_*i*_ = ($x_{i}$, $y_{i}$, $z_{i}$) is the Cartesian coordinates of the *i*-th bead, and *r*_*c*_ = ($x_{\text{c}}$, $y_{\text{c}}$, $z_{\text{c}}$) is that of the center of mass. The scalar radius of gyration is $R_{\text{g}}^{2}=\frac{1}{N}\sum_{i=1}^{N}{\left(r_{i}-r_{\text{c}}\right)}^{2}$.

To quantitatively measure the surface contact, we count the number of contact *N* for each bead *i* in the 3D conformation by using a distance cutoff of 2.5*σ* = 3.7 nm for the pairs of interacting beads. This distance displays a bimodal feature originating from the bonded and non-bonded interaction. The distance between beads *i* and *j* in the reference 2D lattice is defined as *D*_*ij*_, and then the averaged 2D distance of contact is

(Equation 3) $$\alpha_{i}=\frac{\sum_{j}D_{ij}}{N_{i}}\text{,}$$

where the summation is taken over all *N*_*i*_ beads in contact with *i*. The value of *α*_*i*_ is related to the bond length for the planar phase, or the interlayer distance for the contact. The number of beads with contact ($\alpha_{i}\neq 0$) is counted as *M*_*i*_. A localization factor *L*_F_ is then defined as

(Equation 4) $$L_{\text{F}}=\frac{\sum_{i}\alpha_{i}}{M_{i}},$$

where the summation is taken over *M*_*i*_. The distribution of *L*_F_ thus includes the contributions from the intramolecular bonding network and surface contact in the local and long-range modes (Figure 4B).

### Machine learning methods

For unsupervised learning, the K-means algorithm^52^ is used for unsupervised clustering. The number of clusters is set to four considering the 1D cylindrical, 2D flat, and 3D spherical limits, as well as the interphase characteristics. We conduct unsupervised clustering with four to six classes according to the 1D-3D features recognized in the simulation and experimental results. Clustering with more than four classes results in sub-division of the crumpled phase (Figure S9A) or the interphase (Figure S9B), which are named as severe and mild sub-classes. However, these sub-divisions do not show essential difference in the geometry and topology. For example, both severe and mild crumples display features of isotropy and local contact, and the two interphases both demonstrate the nature of transition states with only minor difference in the degree of shrinkage. Therefore, our discussion in this study is focused on the results using four clusters to avoid redundant sub-division or over-refined classification.

2D supervised learning uses the results of unsupervised learning for labeling. The model contains 13 convolutional layers, 1 flattening layer, and 2 fully connected layers. The rectified linear unit (RELU) is used as the activation function except for the last layer, which uses the softmax function. The VGG16 neural network^53^ implemented in TensorFlow^54^ is used to construct the architecture of the convolution layers. The root-mean-square prop (RMSprop) algorithm is used as the optimizer. The learning rate is $2\times 10^{-5}$ without further specification, and the cross-entropy is chosen as the loss function.

3D supervised learning uses the PointNet algorithm implemented in Pytorch for morphological classification.^55^ The model consists of three convolution layers, one maximum pooling layer, and three fully connected layers. The activation function is RELU except for the last layer, which uses the softmax function. The adaptive moment estimation (Adam) and StepLR are used as the optimizer and learning rate scheduler. The negative log likelihood is used as the loss function.

The confusion scheme is used to explore the possible path of transition between the morphological phases.^47^ In this scheme, a value of potential energy (*E*) in the range of $\left[E_{\text{min}},E_{\text{max}}\right]$ is specified to discriminate the morphological data into two classes ($\left[E_{\text{min}},E\right]$ and $\left[E,E_{\text{max}}\right]$). The accuracy of this binary classification through supervised learning is then calculated. The critical energy (*E*_c_) is determined at the local maxima of the accuracy, which may correspond to the transition state between the morphological phases with potential energies lower or higher than *E*_*c*_. On the other hand, the local minima correspond to the well-recognized classes.

The true positives (TP), true negatives (TN), false positives (FP), and false negatives (FN) of the morphological phases are counted to calculate the metrics of accuracy $\left(\frac{\sum_{i}{\text{TP}}_{i}}{\sum_{i}{\text{TP}}_{i}+{\text{FN}}_{i}}\right)$, precision $\left(\sum_{i}{\text{weight}}_{i}\times {\text{precision}}_{i}\right)$, recall $\left(\sum_{i}{\text{weight}}_{i}\times {\text{recall}}_{i}\right)$, and F1 score $\left(\sum_{i}{\text{weight}}_{i}\times \text{F}1{\text{score}}_{i}\right)$, where *i* is the index of morphological phases, and ${\text{weight}}_{i}=\frac{{\text{TP}}_{i}+{\text{FN}}_{i}}{\sum_{i}{\text{TP}}_{i}+{\text{FN}}_{i}}$, ${\text{precision}}_{i}=\frac{{\text{TP}}_{i}}{{\text{TP}}_{i}+{\text{FP}}_{i}}$, ${\text{recall}}_{i}=\frac{{\text{TP}}_{i}}{{\text{TP}}_{i}+{\text{FN}}_{i}}$, $\text{F}1{\text{score}}_{i}=\frac{2\times {\text{precision}}_{i}\times {\text{recall}}_{i}}{{\text{precision}}_{i}+{\text{recall}}_{i}}$.^64^ The weighted average is introduced to account for the class imbalance. The accuracy and recall are the same in this condition. Accuracy reflects the overall predictive power of the model; that is, the proportion of correctly identified phases. Precision measures the exactness of the model predictions through the ratio of correct recognition in the prediction of a certain phase. Recall characterizes the effectiveness of the model to identify positive labels; that is, the ratio of the identified phases in the actual class of certain phases. F1 score is a comprehensive metric considering contributions from both precision and recall. A high F1 score of a model indicates high-precision prediction of morphological phases and complete recognition of conformation. Three independent experiments are performed by varying the training epoch. The mean and standard deviation of metrics of different models are calculated.

## Data Availability

The data used in this study are generated from molecular simulations. The codes and data used in the paper are available at https://zenodo.org/badge/latestdoi/452994542.

## Supplemental information

Document S1. Figures S1–S9 and Tables S1 and S2

Document S2. Article plus supplemental information
