# Supplementary material for: Unraveling the morphological complexity of two-dimensional macromolecules
Source: Patterns (N Y). 2022 Apr 22;3(6):100497. doi: 10.1016/j.patter.2022.100497 (PMC9214330; doi:10.1016/j.patter.2022.100497)
Supplement: Document S1. Figures S1–S9 and Tables S1 and S2 [file mmc1.pdf]

Patterns, Volume *3*

Supplemental information

Unraveling the morphological complexity of two-dimensional macromolecules

Yingjie Zhao, Jiianshu Qin, Shijun Wang, and Zhiping Xu

Supplemental Information

This Supplemental Information Material includes **Supplemental Figures S1-S9 and Tables S1-S2**.

Supplemental Figure S1.

Correlation between features extracted from the 2D map of adhesion energy and morphological parameters extracted from the 3D point set.

Supplemental Figure S2.

The energy landscape of morphological phases.

Supplemental Figure S3.

Morphological classification using 2D energy-map labels.

Supplemental Figure S4.

Test samples that fail in prediction.

Supplemental Figure S5.

Parameter dependence of the sample distribution.

Supplemental Figure S6.

The size effects.

Supplemental Figure S7.

The recognition of morphological phases with defects.

Supplemental Figure S8.

The effect of spatial confinement and environmental factors.

Supplemental Figure S9.

Clustering of morphological phases by specifying more than 4 classes.

Supplemental Table S1.

Evaluation of the statistical learning models.

Supplemental Table S2.

The parameters of coarse-grained (CG) force field for GO.

Supplemental Figure S1

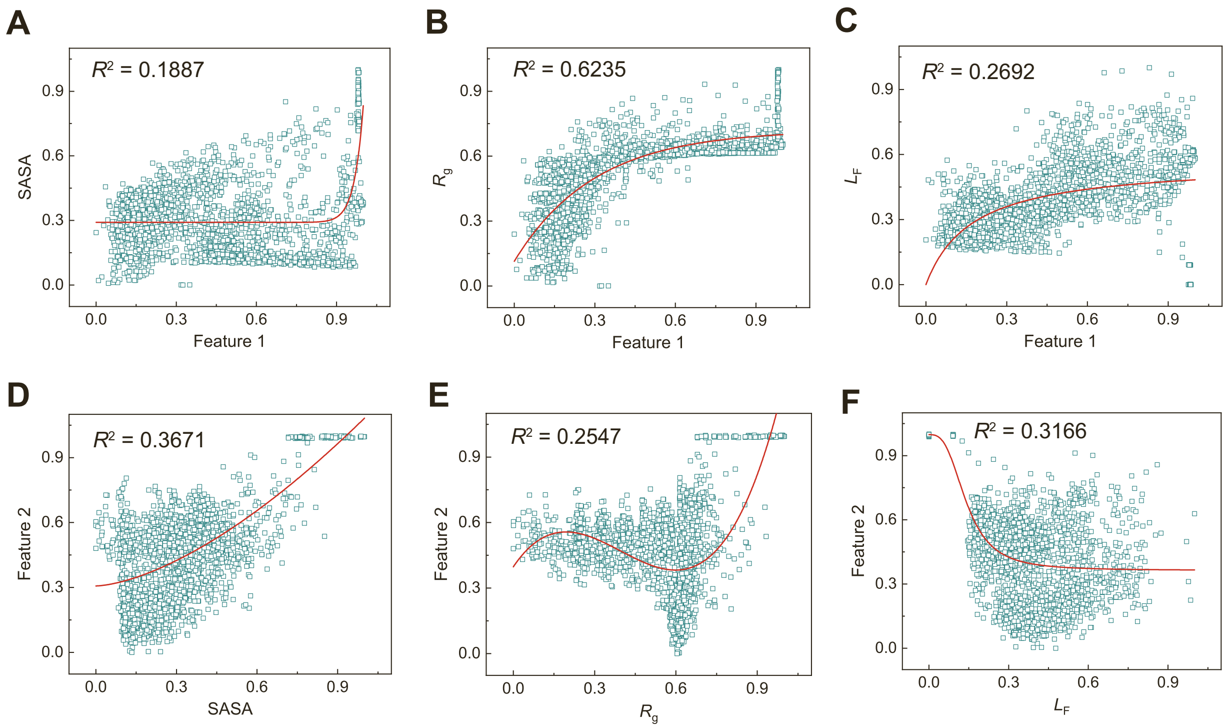

Figure S1 Correlation between features extracted from the 2D map of adhesion energy and morphological parameters extracted from the 3D point set. (**A–C**) The correlation between the first principal feature (P1) and nondimensionalized values of SASA, R_{g}, and L_{F}. (**D–F**) The correlation between the second principal feature (P2) and nondimensionalized values of SASA, R_{g}, and L_{F}.

Supplemental Figure S2

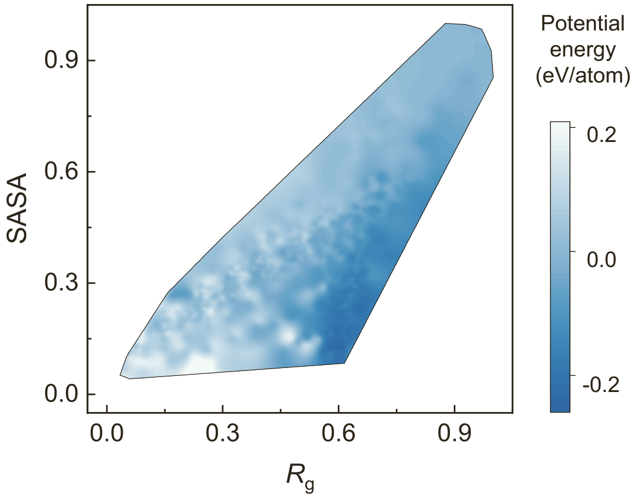

Figure S2 The energy landscape of morphological phases. The crumples and folds own high and low potential energies for their strong lattice distortion and surface adhesion, respectively, and that of the quasi-flat phases or the interphases is located between them.

Supplemental Figure S3

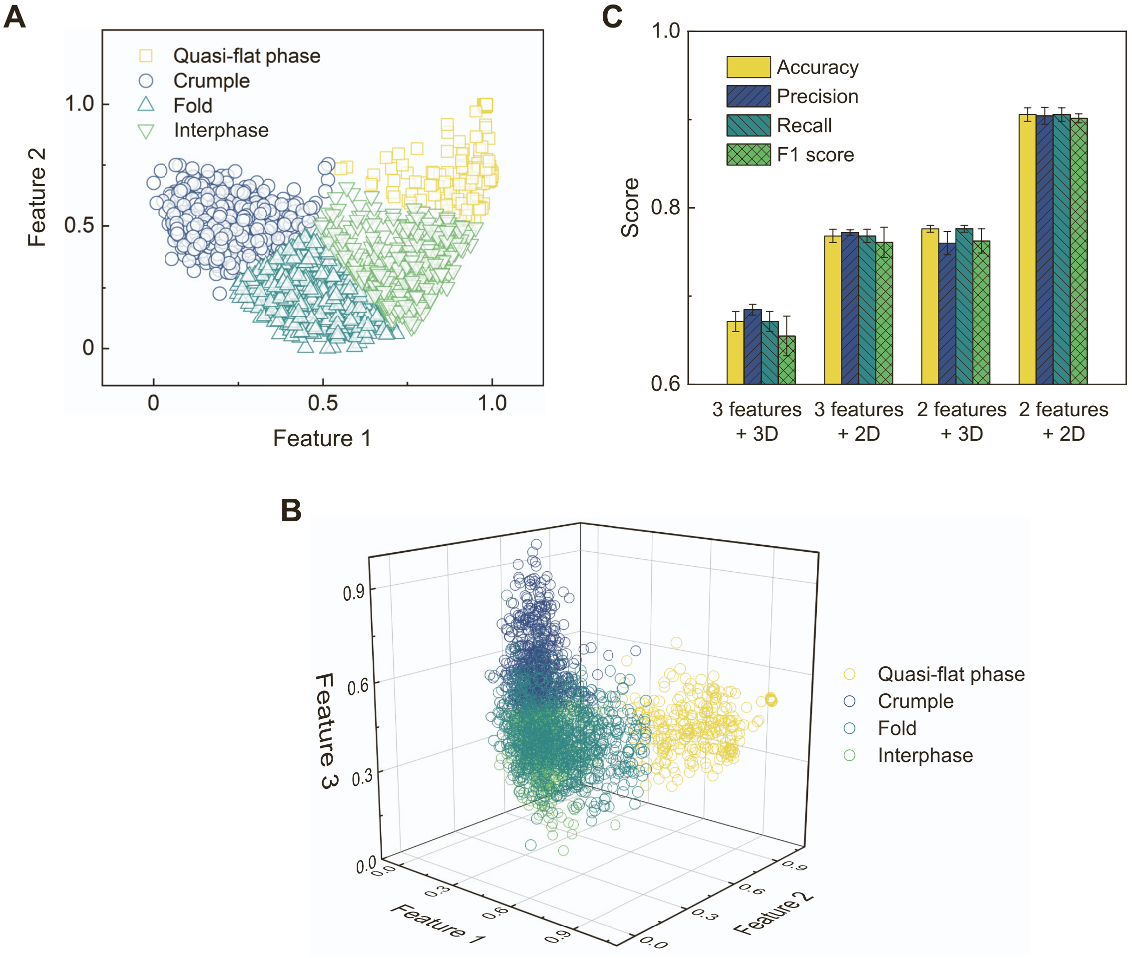

Figure S3 Morphological classification using 2D energy-map labels. (**A**, **B**) Clustering using normalized principal features P1-P2 and P1-P3. (**C**) Evaluation of the statistical learning models.

Supplemental Figure S4

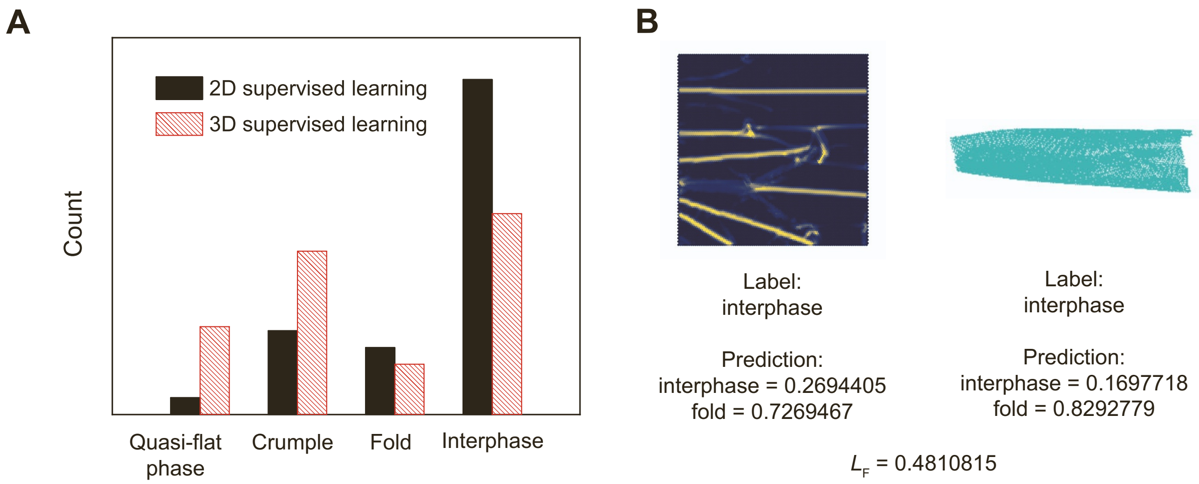

Figure S4 Test samples that fail in prediction. (**A**) The distribution of false negatives for 2D and 3D supervised learning in the quasi-flat, crumpled, folded phases, and the interphases. (**B**) Both 2D and 3D supervised learning yield the same prediction that differs from the labels in unsupervised learning. The calculated value of L_{F} suggests that the prediction of supervised learning is more reasonable than the labels from unsupervised learning.

Supplemental Figure S5

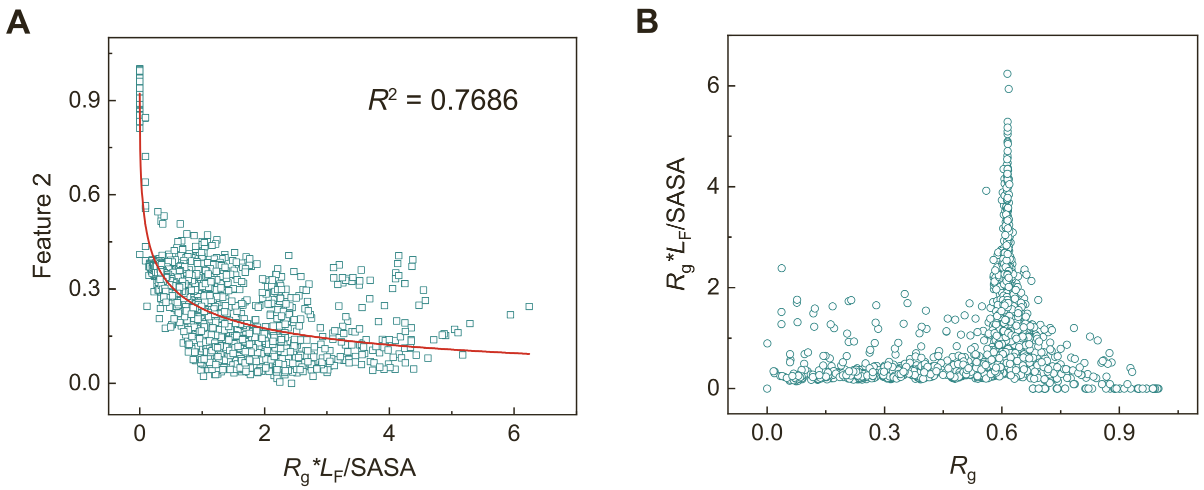

Figure S5 Parameter dependence of the sample distribution. (**A**) The combined parameter $R_g L_F/\mathrm{SASA}$ shows relatively high correlation with the second principal feature extracted from the 2D map of strain energy. (**B**) The distribution of samples in the space spanned by R_g and the combined parameter.

Supplemental Figure S6

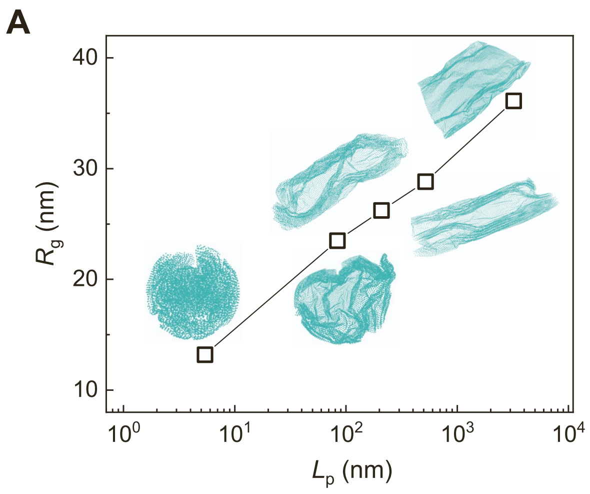

Figure S6 The size effects. (**A**) The radius of gyration (R_g) of 2D macromolecules measured with different persistence length L_p. The initial configuration of graphene is a flat square sheet with a lateral size of $L = 100$ nm. (**B**) The distribution of samples with $L = 50$, 100, and 500 nm. The three limiting phases (1D cylinder, 2D flat, and 3D sphere) of samples with $L = 50$ and 500 nm are plotted with results for $L = 100$ nm.

Supplemental Figure S7

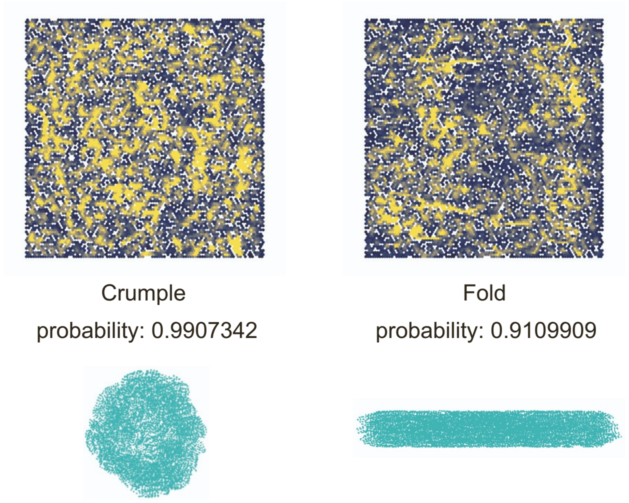

Figure S7 The recognition of morphological phases with defects. The machine learning procedure is applied to 2D macromolecules with 20% vacancies, which create localized lattice distortion and modify the non-bonding interaction, resulting in additional features of geometrical deformation and topological contact.

Supplemental Figure S8

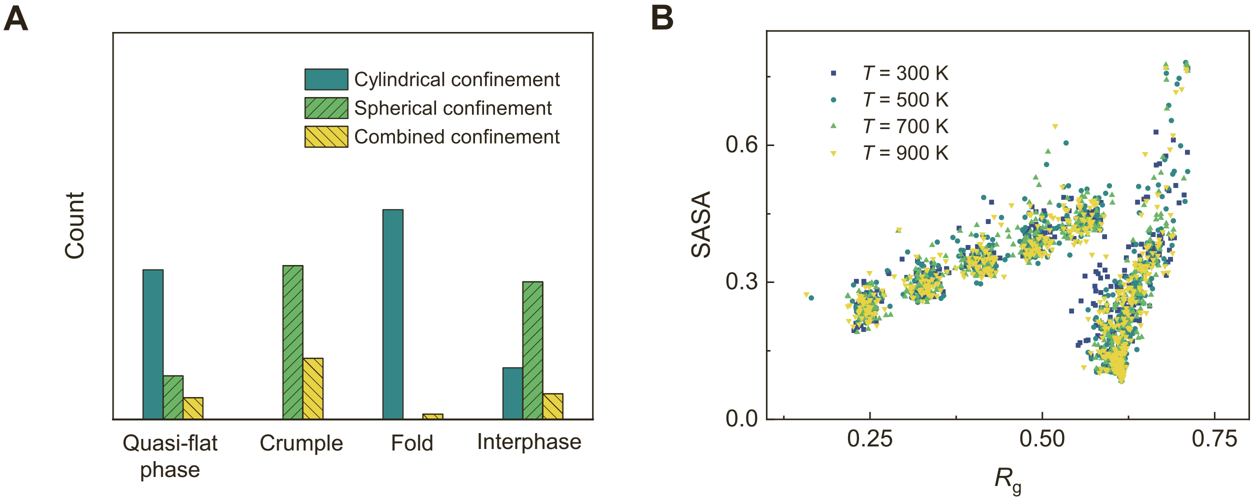

Figure S8 The effect of spatial confinement and environmental factors. (**A**) The count of spatial confinement conditions for the corresponding conformational phases. (**B**) The distribution of the morphological phases show minor temperature dependence at 300 K $-$ 900 K.

Supplemental Figure S9

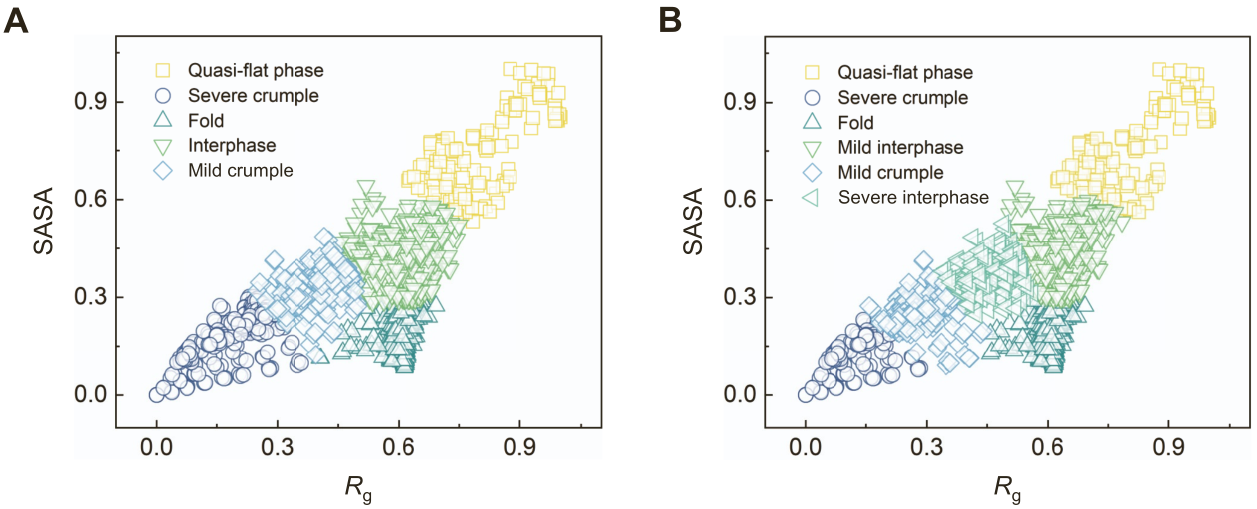

Figure S9 Clustering of morphological phases by specifying more than 4 classes. (**A**) Specifying 5 classes results in severe and mild sub-division of the crumpled phases. (**B**) Specifying 6 classes results in sub-division of the crumpled phase and the interphases.

Supplemental Table S1

Table S1 Evaluation of the statistical learning models

Models	Accuracy	Precision	Recall	F1 score
G	0.8598 ± 0.0097	0.8715 ± 0.0064	0.8598 ± 0.0097	0.8589 ± 0.0077
G+T	0.8832 ± 0.0062	0.8879 ± 0.0034	0.8832 ± 0.0062	0.8798 ± 0.0080
G+P	0.9124 ± 0.0095	0.9135 ± 0.0095	0.9124 ± 0.0095	0.9118 ± 0.0098
G+T+P	0.9421 ± 0.0134	0.9427 ± 0.0127	0.9421 ± 0.0134	0.9420 ± 0.0133
P1-P3+3D	0.6712 ± 0.0114	0.6847 ± 0.0062	0.6712 ± 0.0114	0.6549 ± 0.0224
P1-P3+2D	0.7682 ± 0.0076	0.7720 ± 0.0031	0.7682 ± 0.0076	0.7609 ± 0.0173
P1-P2+3D	0.7763 ± 0.0038	0.7599 ± 0.0132	0.7763 ± 0.0038	0.7625 ± 0.0137
P1-P2+2D	0.9057 ± 0.0076	0.9043 ± 0.0095	0.9057 ± 0.0076	0.9015 ± 0.0050

Supplemental Table S2

Table S2 Parameters of the coarse-grained (CG) force field for 2D macromolecules. The values were taken from reported parameters for GO[1].

Parameters	Units	Values
Equilibrium distance, r_0	Å	10
Equilibrium dihedral angle, φ_0	degree	180
Bond stiffness, k_s	kcal/(mol·Å^2)	120
Dihedral angle stiffness, k_b	kcal/mol	$1-60$
$12-6$ Lennard-Jones parameters, σ	Å	14.8
$12-6$ Lennard-Jones parameters, ε	kcal/mol	1

Supplemental References

1. Wang, Y., Wang, S., Li, P., Rajendran, S., Xu, Z., Liu, S., Guo, F., He, Y., Li, Z., Xu, Z., et al. (2020). Conformational phase map of two-dimensional macromolecular graphene oxide in solution. Matter *3*, 230–245. 10.1016/j.matt.2020.04.023.
